# Ambulance diversion and ED destination by race/ethnicity: evaluation of Massachusetts’ ambulance diversion ban

**DOI:** 10.1186/s12913-022-08358-8

**Published:** 2022-08-03

**Authors:** Amresh D. Hanchate, William E. Baker, Michael K. Paasche-Orlow, James Feldman

**Affiliations:** 1 Department of Social Sciences and Health Policy, Division of Public Health Sciences, Wake Forest School of Medicine, Medical Center Boulevard, Winston-Salem, NC 27157-1063 USA; 2 Section of General Internal Medicine, Boston University School of Medicine, Boston, MA 02118 USA; 3 Department of Emergency Medicine, Boston University School of Medicine, Boston, MA 02118 USA; 4 Boston Medical Center, Boston, MA 02118 USA

**Keywords:** Emergency medical services, ambulance diversion, Emergency department, Disparity, Race, Ethnicity, Safety-net hospital

## Abstract

**Background:**

The impact of ambulance diversion on potentially diverted patients, particularly racial/ethnic minority patients, is largely unknown. Treating Massachusetts’ 2009 ambulance diversion ban as a natural experiment, we examined if the ban was associated with increased concordance in Emergency Medical Services (EMS) patients of different race/ethnicity being transported to the same emergency department (ED).

**Methods:**

We obtained Medicare Fee for Service claims records (2007–2012) for enrollees aged 66 and older. We stratified the country into patient zip codes and identified zip codes with sizable (non-Hispanic) White, (non-Hispanic) Black and Hispanic enrollees. For a stratified random sample of enrollees from all diverse zip codes in Massachusetts and 18 selected comparison states, we identified EMS transports to an ED. In each zip code, we identified the most frequent ED destination of White EMS-transported patients (“reference ED”). Our main outcome was a dichotomous indicator of patient EMS transport to the reference ED, and secondary outcome was transport to an ED serving lower-income patients (“safety-net ED”). Using a difference-in-differences regression specification, we contrasted the pre- to post-ban changes in each outcome in Massachusetts with the corresponding change in the comparison states.

**Results:**

Our study cohort of 744,791 enrollees from 3331 zip codes experienced 361,006 EMS transports. At baseline, the proportion transported to the reference ED was higher among White patients in Massachusetts and comparison states (67.2 and 60.9%) than among Black (43.6 and 46.2%) and Hispanic (62.5 and 52.7%) patients. Massachusetts ambulance diversion ban was associated with a decreased proportion transported to the reference ED among White (− 2.7 percentage point; 95% CI, − 4.5 to − 1.0) and Black (− 4.1 percentage point; 95% CI, − 6.2 to − 1.9) patients and no change among Hispanic patients. The ban was associated with an increase in likelihood of transport to a safety-net ED among Hispanic patients (3.0 percentage points, 95% CI, 0.3 to 5.7) and a decreased likelihood among White patients (1.2 percentage points, 95% CI, − 2.3 to − 0.2).

**Conclusion:**

Massachusetts ambulance diversion ban was associated with a reduction in the proportion of White and Black EMS patients being transported to the most frequent ED destination for White patients, highlighting the role of non-proximity factors in EMS transport destination.

**Supplementary Information:**

The online version contains supplementary material available at 10.1186/s12913-022-08358-8.

## Introduction

Ambulance diversion, the practice by which emergency departments (EDs) are temporarily closed to emergency medical service (EMS) arrivals, and characterized by the Institute of Medicine as “antithetical to quality medical care”, remains common and controversial [1–7]. ambulance diversion has been associated with delayed treatment and adverse outcomes, including higher mortality” [8–11]. There is little experimental evidence on the impact of ambulance diversion, particularly on potentially diverted patients living in urban areas where ambulance diversion is concentrated [8]. On 1/1/2009, Massachusetts became the first and, to date, the only state to ban ambulance diversion across the state. In this study, we treated the Massachusetts ambulance diversion ban as a natural experiment to examine the potential impact of ambulance diversion on ED destination, with a particular focus on differences by patient race/ethnicity [12–14].

While the prior literature has focused on the relationship between ambulance diversion and patient outcomes (e.g., mortality) for high-risk conditions (e.g., trauma), our interest is in key proximate outcomes of EMS transport, including the destination ED, the likelihood of transport to a safety-net ED and transport distance [8–11]. Most EMS transports to an ED – prompted by a 911 call – are not for high acuity life-threatening conditions; as such, an examination of proximate outcomes enables the assessment of the potential impact of ambulance diversion across a wide spectrum of EMS transports and more importantly among socioeconomic subgroups that are more vulnerable to being diverted since ambulance diversion is more prevalent in urban areas [1, 4]. Specifically, we can examine if ambulance diversion influences the likelihood of patients of different race/ethnicity being transported to the same ED as non-Hispanic White patients (i.e., concordance). Evidence indicates that differences in destination EDs and hospitals are associated with racial/ethnic differences in the quality of inpatient care and patient outcomes [15–18].

Using national Medicare claims data, covering adults aged 65 and older, we examined changes in EMS transports following the ambulance diversion ban in Massachusetts and contrasted them with the changes in selected comparison states. As transport patterns are influenced by local geography, availability, and proximity to providers, we compared transports of racial/ethnic minority patients with those of their non-Hispanic White counterparts residing in the same zip code. Based on the premise that the primary determinant of destination ED is proximity, we hypothesized that the ambulance diversion ban would result in a higher proportion of EMS patients from a zip code being transported to the same ED and a narrowing of the differences in this proportion by race/ethnicity (i.e., increased concordance in ED destination) [19]. As a secondary outcome, we also examined the likelihood of transport to a safety-net ED.

## Methods

### Data sources and study cohort

From the national database of Medicare enrollees each year from 2007 to 2012, we selected those aged 66 and older adults with continuous Fee for Service coverage for 3 years or until the date of death (see Supplement Online eTables 1, 2, 3, 4 and 5 for details on the identification of the study cohort) [20]. We stratified all eligible enrollees by their residence zip code (*N* = 38,423 zip codes) into four race/ethnic groups: Hispanics, (non-Hispanic) Blacks, (non-Hispanic) Whites, and others. We identified the subgroup of zip codes with racial/ethnic diversity, defined as containing more than 10 Hispanic, Black, and White enrollees (*N* = 5606 zip codes). For a stratified random sample of enrollees from the diverse zip codes, we obtained healthcare utilization claims data for 2007–2012 (with 1 to 3-year follow-up for each enrollee). We identified all EMS transports to an ED in the sample population and included only zip codes with at least five transports from each of the three race/ethnic groups of interest (*N* = 3953 zip codes). These zip codes are present in almost all states of the country. Since Massachusetts is a predominantly urban state with a denser population, for better comparability with Massachusetts, we identified the subset of states (*N* = 18) with at least 50 zip codes since this excluded states with a higher proportion of the rural population or with no major metropolitan area (see eTable 3). The 3331 zip codes from the selected 18 states and Massachusetts had 27.8% of the overall national eligible enrollee population. We performed a sensitivity analysis using alternative combinations of comparison states (see below). Our study cohort consisted of a (stratified) random sample of 744,791 enrollees residing in the 3331 zip codes.

Using the American Hospital Association annual survey data (2007–2012), we obtained the geographic location of all destination EDs in the Medicare claims data and the proportion of Medicaid patients served [21]. Medicaid is the public coverage for which eligibility is based on low income. We obtained zip code level data on population distribution by race/ethnicity and socioeconomic status from the 2010 decennial census and 2007–2011 American Community Survey from the Census Bureau [22].

### Outcome measures

As the study cohort comes from zip codes across the country with diverse geographic characteristics, a distance-based outcome measure of EMS transport (e.g., miles to destination ED) suffers from limited comparability across areas with large systematic differences in transport distances. We, therefore, base our main outcome on the most frequent destination ED for patients from each zip code grouped by race/ethnicity. Specifically, we identified the most frequent destination ED among White enrollees as the “reference ED” destination for the zip code (see eFig. 1 for a map of reference EDs for zip codes in Boston, Massachusetts). Our main outcome measure was a dichotomous indicator (0/1) of whether each patient EMS transport was to the reference ED in the respective zip code. As a secondary outcome, we also examined whether the destination ED was a safety-net hospital (dichotomous indicator). In defining safety-net hospitals, we obtained the share of all hospial patients who were covered by Medicaid for all hospitals in each region (hospital referral region) and identified the top quartile of hospitals in terms of Medicaid share of patients as safety-net hospitalss [23].

### Covariates

Using the principal diagnosis (ICD-9-CM code) for the ED visit following the incident EMS transport we identified seven conditions with high mortality risk [24]: acute myocardial infarction (AMI), congestive heart failure, pneumonia, stroke, sepsis, gastrointestinal bleeding, and arrhythmia; all other ED visits were grouped as Other [25]. We used the Chronic Condition Data Warehouse classification to identify each of the 23 comorbidity conditions based on prior claims records [26, 27].

### Subgroups

We used the combined race/ethnicity indicator to categorize patients into four groups: Hispanic, (non-Hispanic) Black, (non-Hispanic) White and others. Prior studies have indicated 97% sensitivity in identifying Black and White enrollees, and 77% sensitivity in identifying Hispanic enrollees [28, 29].

We examined the impact of the ambulance diversion ban on several subgroups. We identified advanced life support (ALS) and basic life support (BLS) EMS transports as another acuity indicator. We grouped patients by the type of ED disposition (outpatient discharge, hospitalization); we also separately examined hospitalizations for the seven high acuity admission conditions. As differences in destination ED may be influenced by multiple EDs in the vicinity, we calculated the distance from the centroid of each zip code to each ED and identified the number of EDs within a 3-mile vicinity. We stratified Massachusetts by EMS regions and identified regions with a higher and lower rate of ambulance diversion at baseline [30]. We identified zip codes in Boston and the 15 largest cities in the comparison states. We measured socioeconomic status at the patient level, using an indicator of eligibility for Medicaid (dual coverage) [31] obtained from the claims data, and at the zip code-level using poverty rate and racial/ethnic minority share of census population [22].

### Statistical methods

We used linear probability models with a difference-in-differences specification to estimate the pre- to post-ban change in the likelihood of being transported to a reference ED among EMS transported patients in Massachusetts relative to the change in patients in comparison states [32–34]. As the ban was announced 6 months prior to its implementation (7/3/2008), we identified 1/1/2007 to 6/30/2008 as the pre-ban period, 7/1/2008 to 12/31/2008 as the transition period, and 1/1/2009 to 12/31/2012 as the post-ban period [35]. With transport to reference ED (0/1) as the outcome, we estimated a linear probability model with zip code-level fixed effects and interaction of indicators of Massachusetts patients with the indicators of transition and post-ban periods as the key covariates [36–38]. Other covariates were patient age, sex, principal ED diagnosis, chronic condition comorbidity indicators, Medicaid eligibility, and calendar year indicators. We adjusted for stratification in sampling by using survey weights and stratification indicators. We obtained standard error estimates clustered at the state level and assessed statistical significance at *p* < 0.05 level (see Supplement Online for additional estimation details) [34, 38].

To obtain corresponding estimates by race/ethnicity, we used a modification of the above model by including a three-way interaction of indicators of race/ethnicity, Massachusetts residence, and post-ban period [34]. A similar three-way interaction approach was used for other SES and geographic subgroups. The same specification was used for the secondary outcome, the proportion transported to a safety-net ED. A key assumption of the difference-in-differences design is that the longitudinal trends in the outcomes would have been similar (“parallel”) in Massachusetts and the comparison states were it not for the ambulance ban. Using data for only the pre-ban period, we performed placebo tests of parallel trends for each of the outcome measures, and by race/ethnicity, to evaluate if the longitudinal trends were similar in Massachusetts and the comparison states prior to the ban [33, 34]. All estimation was performed using Stata Version 16.1 [39]. The institutional review board at Wake Forest School of Medicine approved this study. We have followed the Strengthening the Reporting of Observational Studies in Epidemiology (STROBE) guideline in reporting our study findings [40].

We performed sensitivity analyses to examine the robustness of the estimates to the choice of comparison states by using alternative combinations: a) the top 10 states and b) the top 5 states by the number of eligible zip codes for inclusion in the study. To examine for any potential indirect influence of the Massachusetts health reform of 2006–2007, we dropped 2007 EMS transports from our study data and re-estimated the main model estimates [41].

## Results

Our study cohort included 361,006 EMS transports during 2007–2012 from 744,791 Medicare enrollees in 3331 zip codes, with 34.9% of the transports from Massachusetts. Most of the patient demographics and comorbidities were similar in Massachusetts and the comparison states (Table 1 and eTable 6). Boston zip codes accounted for 14.5% of pre-ban transports in Massachusetts, while 14.8% of the transports from the comparison states were from the 15 largest cities.

**Table 1:** Characteristics of EMS Transports: Massachusetts vs. Comparison States, 2007–2012

Characteristic	All	Massachusetts		Comparison states	
	2007–2012	Pre-ban	Post-ban	Pre-ban	Post-ban
Number of ED visits	361,006	33,552	92,723	54,602	149,869
White patients, non-Hispanic	181,337	26,319	72,293	17,362	49,954
Black patients, non-Hispanic	102,121	4173	11,756	21,253	57,417
Hispanic patients	65,889	2029	5886	14,471	38,135
Other patients	10,659	1031	2788	1516	4363
Age, %					
66–74	26.5%	25.0%	27.5%	26.7%	26.8%
75–84	40.8%	41.2%	37.2%	43.6%	39.6%
85+	32.7%	33.9%	35.4%	29.8%	33.6%
Female, %	66.9%	66.7%	67.4%	68.2%	66.4%
Medicaid (dual coverage) eligible, %	27.2%	31%	29.8%	26.4%	27.6%
*Patient status indicators*					
Advanced Life Support (ALS) transports, %	64.9%	53.0%	54.2%	65.1%	65.4%
ED visit resulting in hospital admission, %	55.2%	59.4%	55.6%	57.4%	54.2%
Principal ED diagnosis, %					
Acute myocardial infarction	1.7%	1.9%	1.5%	2.1%	1.6%
Congestive heart failure	3.6%	4.2%	4.1%	3.7%	3.5%
Pneumonia	3.1%	3.8%	3.0%	3.3%	3.0%
Stroke	2.1%	1.7%	1.5%	2.5%	2.0%
Sepsis	3.1%	1.2%	2.2%	2.6%	3.4%
Gastrointestinal bleeding	1.8%	1.5%	1.7%	1.9%	1.8%
Arrhythmia	3.9%	4.4%	3.8%	4.1%	3.8%
Serious injury/trauma	3.9%	3.0%	3.3%	4.4%	3.8%
Other	76.9%	78.3%	78.8%	75.5%	77.2%
*Area characteristics*					
Number of EDs in 3-mile vicinity, %					
0 or 1	82.9%	70.1%	77.9%	82.6%	83.4%
2 or more	17.1%	29.9%	22.1%	17.4%	16.6%
Urban location, No. (%)					
Zip code in largest 16 cities	14.6%	14.5%	14.0%	14.8%	14.6%
Other zip codes	85.4%	85.5%	86.0%	85.3%	85.4%
Zip code households in poverty, %					
Lowest poverty tertile	34.8%	32.6%	34.6%	33.3%	35.4%
Second tertile	32.5%	29.0%	29.9%	32.8%	32.5%
Highest poverty tertile	32.7%	38.4%	35.6%	33.9%	32.1%
Zip codes with > 25% census population black, %	15.8%	10.1%	9.4%	16.3%	15.9%
Zip codes with > 25% census population Hispanic, %	24.5%	13.8%	11.3%	25.7%	24.9%

1) Pre-ban refers to January 1, 2007 to June 30, 2008, and post-ban refers to January 1, 2009 to December 31, 2012

2) Note that the All column includes all EMS transports in the study period. The Massachusetts and Comparison states columns include only the transports during the pre-ban and post-ban periods; the counts from the transition period are not reported. The All column includes all the periods

3) We have reported the number of EMS transports by race/ethnicity to indicate the oversampling of racial/ethnic minorities. All the remaining summary statistics (% distribution) were based on the stratified sampling weights to reflect the characteristics of the underlying eligible Medicare enrollees (approximately 5.5 million each year) from the 3354 zip codes included in the study

4) The largest 16 cities were: Austin, TX; Boston, MA; Chicago, IL; Columbus, OH; Dallas, TX; Houston, TX; Indianapolis, IN, Jacksonville, FL Los Angeles, CA; New York, NY; Philadelphia, PA; Phoenix, AZ; San Antonio, TX; San Diego, CA; San Francisco, CA; San Jose, CA We included only the zip codes within the city (not metropolitan) area

**Fig. 1:**
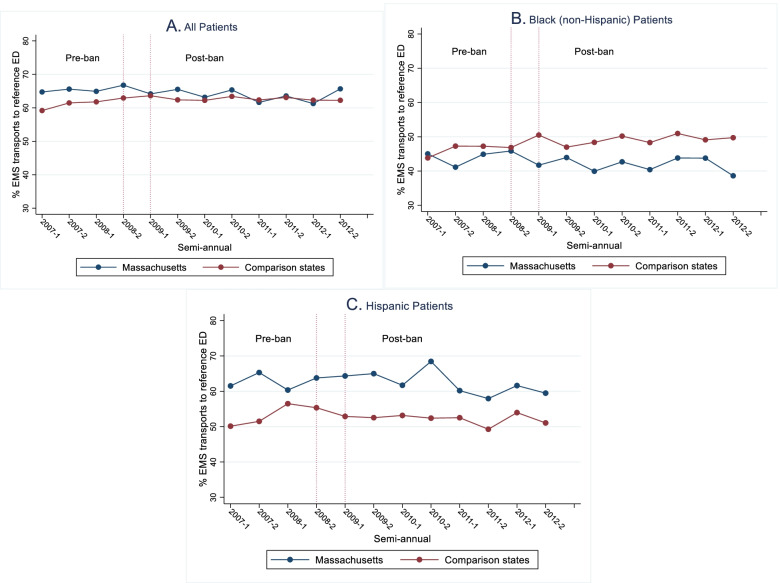
Proportion (%) of EMS Transports to Reference ED: All and Racial/ethnic Minorities

Figure 1 shows that the proportion of all EMS transports to the most frequent ED destination for White patients (i.e., reference ED) is between 60 to 65% in all states and years. The proportion is lower among Black and Hispanic patients. Comparison of the transport distance indicated that the average distance for transports to the reference ED (4.8 miles) was 1.62 miles shorter than that of the second most frequent ED destination (95% confidence interval, 1.58 to 1.66) (eTable 7). This difference was higher in zip codes with fewer EDs in the 3-mile vicinity.

The proportion of all transports to the reference ED decreased from 65.1 to 63.8% between the pre-ban to the post-ban period in Massachusetts; in the comparison states, the proportion increased from 60.9 to 62.7% (Table 2). Adjusted for compositional changes, particularly by area, the ambulance diversion ban was associated with a 2.7 percentage point decrease in Massachusetts in the proportion transported to the reference ED (95% confidence interval, − 4.0 to − 1.4) (eTable 8). Pre-ban, the proportion of Black patients transported to the reference ED was 43.6 and 46.2% in Massachusetts and comparison states, respectively. The ambulance diversion ban was associated with a 4.9 percentage point decrease in the proportion of Black patients transported to the reference ED (95% confidence interval, − 6.2 to − 1.9).

**Table 2:** Change in proportion of EMS transports to reference ED associated with Massachusetts AD ban

Patient cohort	% EMS transports to reference ED in Massachusetts			% EMS transports to reference ED in comparison states			Unadjusted relative change (%)	Adjusted relative change [95% CI]	*p*-value
	Pre-ban	Post-ban	percentage point change	Pre-ban	Post-ban	percentage point change			
All	65.1%	63.8%	−1.3	60.9%	62.7%	1.8	−3.1%	**−2.7 [− 4.0, − 1.4]**	0.001
Race/ethnicity									
White patients, non-Hispanic	67.2%	66.0%	−1.2	64.1%	65.9%	1.8	− 3.0%	**−2.7 [− 4.5, − 1.0]**	0.004
Black patients, non-Hispanic	43.6%	41.8%	−1.8	46.2%	49.3%	3.1	−4.9%	**− 4.1 [−6.2, − 1.9]**	0.001
Hispanic patients	62.5%	62.2%	−0.3	52.7%	52.2%	−0.5	0.2%	1.0 [−1.1, 2.9]	0.359

1) % EMS transports to reference ED in Massachusetts and comparison states are observed measures

2) Unadjusted relative change is the difference in the above %s between Massachusetts and comparison states

3) Adjusted relative change is obtained from the difference-in-differences linear probability models with the dichotmous indicator of transport to reference ED as the outcome. A separate model (two-way difference-in-differences) for “All”. A separate three-way difference-in-differences model was estimated for the estimates by race/ethnicity. See Online Supplement for the estimation model details and eTable 8 for the full model estimates

Table 3 provides the corresponding estimates for a range of subgroups based on indicators of patient acuity, geographic location, and socioeconomic status. Broadly we find similarity in the estimates of change in the proportion transported to the reference ED associated with ambulance diversion ban. In the Massachusetts regions with higher (pre-ban) diversion rates, the proportion of patients brought to the reference ED decreased by 2.9 percentage points (95% confidence interval, − 4.4 to − 1.3); in the regions with lower diversion rates, the change was not significant. Using transports in Boston and the 15 largest cities in the comparison states indicated a reduction of 2.5 percentage points (95% confidence interval, − 4.9 to − 0.3) in the proportion transported to the reference ED. Grouping zip codes by poverty tertiles also indicated similar changes across poverty groups.

**Table 3:** Change in proportion of EMS transports to reference ED associated with Massachusetts AD ban: Sub-groups

Patient cohort	# EMS transports	% EMS transports to reference ED in Massachusetts			% EMS transports to reference ED in comparison states			Unadjusted relative change (percentage points)	Adjusted relative change (percentage points) [95% CI]	*p*-value
		Pre-ban	Post-ban	% point change	Pre-ban	Post-ban	% point change			
*Patient acuity subgroups*										
Type of EMS transport										
Advanced Life Saving (ALS)	213,171	68.1%	67.3%	−0.8	61.6%	63.3%	1.7	−2.5	**−2.8 [−4.7, −1.0]**	0.005
Basic Life Support (BLS)	147,835	61.7%	59.7%	−2.0	59.5%	61.3%	1.8	−3.8	**−3.2 [−4.9, −1.6]**	0.001
Disposition from ED										
Outpatient discharge	159,135	65.2%	63.8%	−1.4	61.7%	63.8%	2.1	−3.5	**−1.7 [− 3.0, −0.3]**	0.02
Hospitalization	201,871	65.0%	63.8%	−1.2	60.3%	61.7%	1.4	−2.6	**−3.0 [−4.7, −1.2]**	0.002
Hospitalization for a high-risk condition	69,137	65.7%	63.8%	−1.9	61.7%	62.5%	0.8	−2.7	−2.0 [−4.7, 0.7]	0.132
*Geographic factors*										
Massachusetts EMS region										
Higher diversion rate regions	307,618	57.3%	56.9%	−0.4	60.9%	62.7%	1.8	−2.2	**−2.9 [−4.4, −1.3]**	< 0.001
Other regions	276,780	74.5%	72.5%	−2.0	60.9%	62.7%	1.8	−3.8	−2.5% [−5.8, 0.8]	0.142
# EDs in 3-mile vicinity										
0 or 1	267,308	70.9%	68.0%	−2.9	63.3%	65.0%	1.7	−4.6	**−2.6 [−4.9, −1.2]**	0.001
2 or more	93,698	51.4%	49.0%	−2.4	49.3%	50.9%	1.6	−4.0	**−2.9 [−5.6, −0.2]**	0.037
Urban location, %										
Zip code in largest 16 cities	74,464	38.6%	39.2%	0.6	44.5%	46.6%	2.1	−1.4	**−2.5 [−4.9, −0.3]**	0.032
Other zip codes	286,542	69.6%	67.8%	−1.8	63.7%	65.4%	1.8	−3.5	**−2.7 [−4.1, −1.3]**	0.001
*Socioeconomic status subgroups*										
Medicaid (dual) coverage										
With Medicaid coverage	153,522	61.7%	61.7%	0.0	56.9%	57.6%	0.7	−0.7	−1.8 [−3.83–0.3]	0.022
Without Medicaid coverage	207,484	66.6%	64.7%	−1.9	62.3%	64.6%	2.3	−4.2	−3.6 [−6.0, −2.1]	< 0.001
Zip code poverty rate										
Lowest poverty tertile	112,254	65.0%	64.2%	−0.8	62.9%	64.8%	1.9	−2.7	**−2.8 [−5.4, − 0.1]**	0.04
Middle poverty tertile	98,335	71.8%	69.7%	−2.1	63.4%	63.5%	0.1	−2.2	**−3.0 [−5.5, −0.5]**	0.023
Highest poverty tertile	150,361	60.1%	58.6%	−1.5	56.4%	59.5%	3.1	−4.6	**−2.7 [−4.2, − 1.1]**	0.002
Zip codes with > 25% census population black, %	69,383	42.6%	41.3%	−1.3	52.5%	54.7%	2.1	−3.4	−1.3 [−4.0, 1.3]	0.297
Zip codes with > 25% census population Hispanic, %	111,757	67.2%	65.7%	−1.5	57.6%	58.8%	1.2	−2.7	**−4.0 [−5.2, − 2.8]**	< 0.001

1) % EMS transports to reference ED in Massachusetts and comparison states are observed measures

2) Unadjusted relative change is the difference in the above %s between Massachusetts and comparison states

3) Adjusted relative change is obtained from 3-way difference-in-differences linear probability models with the dichotmous indicator of transport to reference ED as the outcome. A separate model for each set of subgroups (e.g., Type of EMS transport). See Online Supplement for the estimation model details

The proportion of EMS transports to a safety-net ED was higher among Black and Hispanic patients, relative to White patients, in Massachusetts and comparison states (Table 4). The ambulance diversion ban was associated with a 3.0 percentage point increase in the proportion transported to a safety-net ED among Hispanic patients (95% confidence interval, 0.3 to 5.7) and a 1.2 percentage point reduction in the proportion among White patients (95% confidence interval, − 2.3 to − 0.2). There was no corresponding change among Black patients.

**Table 4:** Change in proportion transported to a safety-net associated with Massachusetts AD ban

Patient cohort	% to safety-net ED in Massachusetts			% to safety-net ED in comparison states			Unadjusted relative change (percentage points)	Adjusted relative change (percentage points) [95% CI]	*p*-value
	Pre-ban	Post-ban	percentage point change	Pre-ban	Post-ban	percentage point change			
All	26.8%	24.4%	−2.4	18.3%	17.9%	−0.4	−2.0	−0.8 [− 1.6, 0.1]	0.090
Race/ethnicity									
White patients, non-Hispanic	24.3%	21.6%	−2.7	16.1%	16.1%	0.0	−2.7	−1.2 [− 2.2, 0.2]	0.150
Black patients, non-Hispanic	53.0%	51.2%	−1.8	28.7%	25.7%	−3.0	1.2	−0.8 [−1.4, 2.9]	0.492
Hispanic patients	36.9%	36.7%	−0.2	27.1%	24.9%	−2.2	2.0	**3.0 [0.3% 5.7]**	0.031

1) % transported to a safety-net ED in Massachusetts and comparison states are observed measures

2) Unadjusted relative change is the difference in the above %s between Massachusetts and comparison states

3) Adjusted relative change is obtained from the difference-in-differences linear probability models with the dichotmous indicator of transport to a safety-net ED as the outcome. A separate model (two-way difference-in-differences) for “All”. A separate three-way difference-in-differences model was estimated for the estimates by race/ethnicity

In testing the key assumption of parallel trends, the placebo tests data for the pre-ban period indicate that longitudinal trendswere similar in Massachusetts and the comparison states for all three outcomes (eTable 9). An exception was for the proportion transported to a reference ED among Hispanics, for whom we found a decreasing trend in Massachusetts prior to the ban. In the sensitivity analysis, the estimates remained consistent (a) using the top 10 comparison states, (b) using only the top 5 comparison states, and (c) excluding 2007 cases for potential confounding with Massachusetts health reform (eTables 10, 11 and 12). The only change was in the latter case, wherein we found an increase among Hispanics in the proportion transported to the reference ED.

## Discussion

Using a Medicare enrollee cohort and an experimental difference in differences study design we estimated the changes in EMS transport outcomes associated with the Massachusetts ban on ambulance diversion in 2009. Focusing on potential changes in the destination ED following the ban, particularly among racial/ethnic minorities, we identified the most common (modal) ED destination among White patients in each zip code as the reference ED, and measured the change in the proportion of co-located patients transported to the reference ED. We found that, prior to the ban, the proportion of non-Hispanic White patients in Massachusetts transported to the reference ED was 67%, and this proportion was smaller among non-Hispanic Black (44%) and Hispanic (63%) patients. The ban was associated with a decrease in the proportion transported to the reference ED among White (2.7 percentage points) and Black (4.1 percentage points) patients, and no change among Hispanic patients. Similar analysis of the proportion of patients co-located in the same zip code transported to a safety-net ED, at baseline, was higher among Black (53%) and Hispanic (37%) patients, relative to White patients (24%). The ban was associated with an increase in the proportion transported to a safety-net ED among Hispanic patients (3.0 percentage points), a reduction among White patients (1.2 percentage points) and no change among Black patients.

Counter to our hypothesis, we found that the ambulance diversion ban in Massachusetts was associated with reduced concordance of EMS transports to EDs. Among White and Black patients, fewer transports were to the reference ED. The pattern of increased dispersion in destination ED was consistent across a wide range of subgroups by patient acuity, sociodemographic characteristics and geographic features. The ban was associated with reduced concordance for EMS transports in Boston and in areas with (baseline) higher diversion rate.

To our knowledge, no previous study has examined the association between the Massachusetts ambulance diversion ban and EMS transport outcomes for potentially diverted patients. One study that focused on the impact within EDs found that the ban was not associated with any change in the length of stay or turnaround time for patients in Boston [35]. Our findings complement the broader literature on the association between ambulance diversion (measured by hours of ED closure) and outcomes (mortality) of patients transported by EMS [8–11, 42, 43]. As these studies are based on observational data without an experimental study design, a limitation is that since ED closures are not randomly determined, ambulance diversion may be correlated with unobserved factors (e.g., ED crowding), which may also affect patient outcomes [8, 43, 44]. Nevertheless, the consistency of the findings of adverse patient outcomes associated with higher ambulance diversion volume (hours) across diverse geographic regions merits consideration. Generally, the adverse patient outcomes from ambulance diversion were attributed to delays in patient transport, although these studies lacked data on transport time or distance. Our study suggests that the important intermediate factor may be the ED/hospital destination rather than transport delays. In our data, the additional travel distance between the first and second most common ED destinations was 1.62 miles overall and 0.88 miles in major cities. Evidence from a recent study on transport times during “diversions” arising from street closures during major marathons resulted in a 4.4-minute longer transport time (and no significant difference in distance transported) [45]. It is unclear if added distance or delays of these magnitudes are associated with adverse patient outcomes, even for high-acuity life-threatening conditions (AMI or stroke). Instead, there is considerable evidence of systematic differences in hospital performance and associated disparities in patient outcomes [15–18, 46, 47].

The finding of reduced concordance in destination ED after the ambulance diversion ban has implications for our understanding of the factors motivating the EMS transport destination. Our hypothesis of increased concordance from the ambulance diversion ban was based on the assumption of proximity as the primary determinant of ED destination. Transport distance to the reference ED is significantly shorter than that to the second most common ED destination. As such, the finding of reduced concordance following the ambulance diversion ban indicates that factors other than proximity may be important determinants of ED destination. Newgard et al. examined data for 176,981 trauma patient transports from 61 EMS providers in western US and found that the most frequent reasond for destination ED were patient or family choice (50.6%), closest facility (20.7%) and specialty resource center (15.2%) [48]. Patients may prefer to be transported to the hospital with prior healthcare use (“home hospital”). Our finding of an increase among Hispanic patients and a decrease among White patients in the proportion transported to safety-net hospitals following ambulance diversion ban is also consistent with minority patients more likely to use safety-net hospitals as their home hospital. At baseline in Massachusetts, the proportion of patients (co-located in the same zip code) transported to a safety-net ED was higher among Hispanic (37%) and Black patients (53%) than among White patients (24%). Transport patterns may also vary systematically across EMS providers [49]. While the literature on ambulance diversion is largely silent on this issue, recognition of other motivations should be taken into account. It suggests that bypassing of the nearest EDs may be more common and results from not only ambulance diversion but also other factors.

## Limitations

We recognize several limitations of the study. First, our identification of change in ambulance diversion is based on the pre- vs. post-ban comparison between Massachusetts and other states. Other contemporaneous changes in Massachusetts, not affecting other states, may confound our findings. Of particular significance is the Massachusetts health reform that expanded Medicaid and insurance coverage. Although this reform only targeted those aged 18 to 64, there may be indirect effects on Medicare patients 65 and older. The Medicaid expansion component became effective in June 2006, and other elements enabling subsidized private coverage were introduced in early 2007 [41]. We performed sensitivity analysis by excluding 2007 data and found the resulting estimates remained consistent. Sensitivity analyses also indicated that alternative choice of comparison states did not affect the results. Second, due to limitations of the claims data, we used the residence zip code to define the destination ED outcome, which may lead to measurement error if the pick-up location is outside the zip code. A national study of EMS transports found that the pick-up location is the patient residence for 80% of transports for those aged 65–84 and 85% of transports for those aged 85 and older [50]. Our difference in differences study design identifies changes occurring after the ambulance diversion ban; therefore, to the extent that the rate of transports from residence are similar in Massachusetts and other states and did not change after ambulance diversion ban, our resulting estimates are unlikely to be confounded. Third, the claims data do not adequately differentiate patient differences in symptoms and severity that may lead to transport to alternative hospitals (based on the capability of services). However, if severity differences between groups do not change between pre- and post-ban periods, then the estimates are robust to the unobserved differences in severity. Fourth, our estimates are based on the Massachusetts experience, and therefore the generalizability of the findings to other geographic regions needs to be assessed. To date, no other states have stopped ambulance diversion. Our data examines those aged 66 and older, and therefore our findings may not be generalizable to the younger population. This limitation arises from the absensce of a national healthcare utilization database in the US covering all ages.

## Conclusions

Our study indicates that the diversion ban in Massachusetts was associated with reduced concordance in the destination ED among White and Black patients. The proportion of patients transported to a safety-net ED also experienced mixed patterns: an increase among Hispanic patients, a decrease among White patients, and no change among Black patients. These findings suggest that EMS transport to the nearest ED may not be the predominant driver of EMS transport destination; instead, patient or EMS provider preferences may also be important factors.

## Supplementary Information

**Additional file 1: eTable 1.** Counts of Medicare enrollees, 2007–2012. **eTable 2.** Racial/ethnic composition of eligible Medicare enrollees. **eTable 3.** Composition of eligible enrollees from zip codes with racial/ethnic diversity, 2009. **eTable 4.** Sample size by year and follow-up cohort composition (zip codes with diversity). **eTable 5.** Sampling design. **eFigure 1.** Most frequent ED/hospital among EMS transported White patients in each zip code in Boston. **eTable 6.** Prevalence of chronic conditions at baseline. **eTable 7.** Comparison of average distance between first and second most frequent destination. **eTable 8.** Estimates of the impact of ban on transport to reference ED: All and by race/ethnicity. **eTable 9.** Parallel trends test results. **eTable 10.** Sensitivity 1 - Select only the top 10 states. **eTable 11.** Sensitivity 2 - Select only the top 5 states. **eTable 12.** Sensitivity 3 - Exclude 2007 cases.

## Data Availability

The data used for this study are from the Centers for Medicare and Medicaid Services (CMS) Fee for Service claims databases under a data use agreement. This agreement restricts the sharing of the data with other researchers.
