# Supplementary material for: Ambulance diversion and ED destination by race/ethnicity: evaluation of Massachusetts’ ambulance diversion ban
Source: BMC Health Serv Res. 2022 Aug 3;22:987. doi: 10.1186/s12913-022-08358-8 (PMC9347077; doi:10.1186/s12913-022-08358-8)
Supplement: Supplementary file 1 — Additional file 1: eTable 1. Counts of Medicare enrollees, 2007–2012. eTable 2. Racial/ethnic composition of eligible Medicare enrollees. eTable 3. Composition of eligible enrollees from zip codes with racial/ethnic diversity, 2009. eTable 4. Sample size by year and follow-up cohort composition (zip codes with diversity). eTable 5. Sampling design. eFigure 1. Most frequent ED/hospital among EMS transported White patients in each zip code in Boston. eTable 6. Prevalence of chronic conditions at baseline. eTable 7. Comparison of average distance between first and second most frequent destination. eTable 8. Estimates of the impact of ban on transport to reference ED: All and by race/ethnicity. eTable 9. Parallel trends test results. eTable 10. Sensitivity 1 - Select only the top 10 states. eTable 11. Sensitivity 2 - Select only the top 5 states. eTable 12. Sensitivity 3 - Exclude 2007 cases. [file 12913_2022_8358_MOESM1_ESM.docx]

**Supplement Online Content**

**Ambulance Diversion and ED Destination by Race/Ethnicity: Evaluation of Massachusetts' Ambulance Diversion Ban**

**Table of Contents**

|  | Section | Page # |
| --- | --- | --- |
| A | Sampling universe and study cohort | 1-7 |
| B | Estimation model | 8 |
| C | Boston: Map of most frequent ED destination (eFigure 1) | 9 |
| D | Prevalence of chronic conditions (eTable 6) | 10 |
| E | Average distance between first and second most ED destination (eTable 7) | 11 |
| F | Full regression model estimates (eTable 8) | 12-14 |
| G | Parallel trends test results (eTable 9) | 15 |
| H | Sensitivity model results (eTables 10-12) | 16-18 |
| I | References | 19 |

**1. Sampling Universe**

*Eligible enrollees:* Our study sample was drawn from the universe of Medicare enrollees during 2007-2012 (N=46.7 million in 2007 to 53.6 million in 2012); a sample was drawn from the universe for each year separately (eTable 1). For this study we selected those aged 66 and older and have continuous Fee for Service (FFS) coverage; for enrollees from 2007 to 2010, we required continuous coverage for 3 years, for enrollees from 2011 we required continuous coverage for 2 years and for enrollees from 2012 we required continuous coverage for 1 year. eTable 2 gives the counts for the selected subgroups for 2007 and 2012, overall and by race/ethnicity.

*Zip codes with race/ethnic diversity:* Using the residence zip code (reported in the Medicare beneficiary files) of continuously FFS enrolled members for each year, we stratified all zip codes by racial/ethnic diversity; a zip code was categorized as diverse if it was the residence zip code of >10 white, >10 black and >10 Hispanic eligible enrollees. Appendix eTable 3 gives the composition of zip codes by racial/ethnic diversity for the 2009 study-eligible sampling universe; these figures were similar for the other years. An additional requirement of the study zip codes was that there be at least 5 EMS transports (to ED) for each of the three race/ethnic groups (from each zip code) during the study period. Finally, we retained only the zip codes in Massachusetts and 18 comparison states with 50 or more zip codes that qualified based on racial/ethnic diversity and at least 5 EMS transports.

The above table identifies qualifying 3,331 zip codes in 2009 data; similar analysis for all years identified a total of 3,354 unique zip codes, which formed the eligible zip codes for this study.

**2. Sample Size**

From the universe of eligible Medicare enrollees from the selected zip codes with race/ethnic diversity we obtained stratified random samples separately for each year. As our focus is on comparing Massachusetts vs. other states, we oversampled from the zip codes in Massachusetts. Within each state group (Massachusetts and Other states) we sampled based on the zip code population size, with larger samples from larger zip codes (see section 3 below). This sampling strategy was applied to the cohort for each year. Medicare utilization records were only obtained for the sample cohorts. Following the rolling cohort design of national surveys (AHRQ's Medicare Expenditure Panel Survey and CMS' Medicare Current Beneficiary Survey), we stratified the sample for each year into three cohorts with each cohort followed for 1 to 3 years.^1-3^ eTable 4 identifies the counts and follow-up periods for the 8 distinct cohorts in the study. Cohort 1 consisted of 255,918 enrollees sampled from the eligible universe in 2007; we obtained utilization records for 1 year (i.e., 1 year of follow-up). Cohorts 2 and 3 was also obtained from the 2007 universe but with 2- and 3-year follow-up, respectively. Cohort 4 is introduced in 2008, based on the 2008 universe, and replaces the retired Cohort 1. Cohorts 3 to 6 have 3 follow-up years. Cohort 7 is followed for 2 years and Cohort 8 for 1 year. Enrollees may be re-sampled in a subsequent cohort if they are no longer in an actively followed cohort. While the total sample count is 1,048,960, after excluding those re-sampled in multiple rounds, the number of unique enrollees was 864,750. After inclusion of zip codes from Massachusetts and the selected 18 comparison states, the unique enrollee count was 744,791; we have reported this figure as the underlying sample size for all the ED visits examined in this study. For more about re-sampling, particularly from Massachusetts, see section 3 below.

**3. Sampling by co-location**

The desired sample count from the universe of each year was obtained by random sampling of enrollees stratified by zip code and race/ethnicity. Following were the sampling criteria.

1) Our preference was to obtain equal number of the 3 race/ethnic groups from each zip code; however, zip codes with racial/ethnic diversity varied considerably in the number of enrollees that could be sampled. The number that can be sampled is given by the number of the smallest race/ethnic group in the zip code; for instance, if a zip code has 100 white enrollees, 20 black enrollees and 12 Hispanic enrollees, then we can sample at most 12 members of each group from the zip code. For better representation of the overall population we chose larger sample sizes from zip codes with larger number of enrollees that could be sampled.

2) The study data was designed as part of a larger study aimed at comparing ED use changes in Massachusetts vs. the remaining states in the country. Therefore, we over-sampled Massachusetts enrollees; approximately one-third of the total sample was from Massachusetts by design. In particular, for many zip codes, we were more likely to select all the black or Hispanic enrollees from the zip codes; consequently, these enrollees were likely to be re-enrolled in a later cohort.

eTable 5 provides the sampling rates by zip code categories: those with >100 enrollees from each race/ethnic group (category 1); those with 26 to 100 enrollees from each race/ethnic group (category 2) and those with 11 to 25 enrollees from each race/ethnic group (category 3). It presents the sampling numbers in Massachusetts and rest of the country separately.

**4. Sampling weights**

Our sampling strategy amounts to stratification of all eligible Medicare enrollees in these zip codes at two levels, first, by zip code, and second, by race/ethnicity. That is, all eligible enrollees in each zip code are stratified into four groups by race/ethnicity: Hispanics, (non-Hispanic) blacks, (non-Hispanic) whites and Others (all the remaining groups combined). As sampling of enrollees was done randomly from each group, the sampling probability for each selected enrollee is given by the ratio between the sample size and the total number of eligible enrollees from the racial/ethnic group in the zip code. The sampling weight is the inverse of this ratio.

Application of sampling weights leads to estimates generalizable to approximately 5.5 million underlying study-eligible Medicare enrollee population each year. This cohort is detailed in eTable 3 for 2009; there were 5,567,122 enrollees in the study-eligible zip codes.

eTable 1. Counts of Medicare enrollees, 2007-2012

| Year | # Medicare enrollees | # Medicare enrollees 66+ continuously enrolled in FFS during same year and following 2 years |
| --- | --- | --- |
| 2007 | 46,694,639 | 20,050,727 |
| 2008 | 47,850,425 | 20,027,650 |
| 2009 | 48,922,869 | 20,249,187 |
| 2010 | 50,088,947 | 20,395,539 |
| 2011 | 51,717,260 | 22,376,862 |
| 2012 | 53,597,183 | 24,658,341 |

Notes:

1) Abbreviation: Fee For Service, FFS

2) For 2011, the last column gives the count of Medicare enrollees continuously enrolled in FFS during 2011-2012

3) For 2012, the last column gives the count of Medicare enrollees continuously enrolled in in FFS during 2012

eTable 2. Racial/ethnic composition of eligible Medicare enrollees

| Year | Medicare enrollee subgroup |  | Race/ethnic group, % | | | |
| --- | --- | --- | --- | --- | --- | --- |
|  |  | Total # enrollees | White enrollees | Black enrollees | Hispanic enrollees | Other enrollees |
| 2007 | All Medicare enrollees | 46,694,639 | 78.01 | 9.91 | 7.76 | 4.32 |
|  | # Medicare enrollees 66+ continuously enrolled in FFS during same year and following 2 years | 20,050,727 | 85.02 | 6.87 | 4.33 | 3.78 |
| 2012 | All Medicare enrollees | 53,597,183 | 76.59 | 10.16 | 8.55 | 4.69 |
|  | # Medicare enrollees 66+ continuously enrolled in FFS during same year | 24,658,341 | 83.63 | 7.45 | 5.04 | 3.88 |

Notes;

1) Abbreviation: Fee For Service, FFS

eTable 3. Composition of eligible enrollees from zip codes with racial/ethnic diversity, 2009

| Zip code category | # zip codes | All enrollees | White enrollees | Black enrollees | Hispanic enrollees |
| --- | --- | --- | --- | --- | --- |
| All unique Medicare enrollees aged 66+ with continuous FFS coverage | 38,423 | 20,249,187 | 17,221,032 | 1,410,898 | 930,727 |
| Subgroup of enrollees in zip codes with racial/ethnic diversity (>10 White, Black and Hispanic enrollees each) | 5,606 | 9,562,563 | 7,676,317 | 791,592 | 624,285 |
| Subgroup of enrollees in zip codes with racial/ethnic diversity & 5 or more EMS trips in study data | 3,953 | 6,934,344 | 5,484,597 | 616,343 | 505,735 |
| Subgroup of enrollees in Massachusetts and 18 comparison states selected on criteria that the state contains at least 50 zip codes with racial/ethnic diversity and 5 or more EMS trips | 3,331 | 5,567,122 | 4,621,602 | 519,362 | 426,158 |

Notes:

1) Abbreviations: Fee For Service, FFS; Emergency Medical Services, EMS

2) The 18 comparison states were: AZ, CA, CT, FL, GA, IL, LA, MI, NC, NJ, NV, NY, OH, OK, PA, TX, VA and WA.

eTable 4. Sample size by year and follow-up cohort composition (zip codes with diversity)

| Cohort |  | | | | | |  |
| --- | --- | --- | --- | --- | --- | --- | --- |
|  | 2007 | 2008 | 2009 | 2010 | 2011 | 2012 |  |
| 1 | 255,918 | 0 | 0 | 0 | 0 | 0 | 255,918 |
| 2 | 109,347 | | 0 | 0 | 0 | 0 | 109,347 |
| 3 | 108,432 | | | 0 | 0 | 0 | 108,432 |
| 4 | 0 | 130,264 | | | 0 | 0 | 130,264 |
| 5 | 0 | 0 | 107,050 | | | 0 | 107,050 |
| 6 | 0 | 0 | 0 | 112,378 | | | 112,378 |
| 7 | 0 | 0 | 0 | 0 | 119,481 | | 119,481 |
| 8 | 0 | 0 | 0 | 0 | 0 | 106,090 | 106,090 |
| All | 473,697 | 130,264 | 107,050 | 112,378 | 119,481 | 106,090 | 1,048,960 |

eTable 5. Sampling design

| Zip code category |  | Number of enrollees sampled from each zip code | | | |
| --- | --- | --- | --- | --- | --- |
|  |  | White enrollees | Black enrollees | Hispanic enrollees | Others |
| *All states except Massachusetts* | | | | | |
| 1. Zip codes with >100 enrollees of each of the 3 race/ethnic groups |  | 14 to 22 | 14 to 22 | 14 to 22 | 1 to 2 |
| 2. Zip codes with 26 to 100 enrollees of each of the 3 race/ethnic groups |  | 6 to 10 | 6 to 10 | 6 to 10 | 1 |
| 3. Zip code with 11 to 25 enrollees of each of the 3 race/ethnic groups |  | 3 to 6 | 3 to 6 | 3 to 6 | 1 |
| *Massachusetts* | | | | | |
| 1. Zip codes with >100 enrollees of each of the 3 race/ethnic groups |  | 1000 to 1500 | 60 to 100 | 60 to 100 | 30 to 50 |
| 2. Zip codes with 26 to 100 enrollees of each of the 3 race/ethnic groups |  | 414 to 690 | 28 to 46 | 28 to 46 | 14 to 23 |
| 3. Zip code with 11 to 25 enrollees of each of the 3 race/ethnic groups |  | 153 to 255 | 10 to 17 | 10 to 17 | 5 to 9 |

**5. Estimation**

We estimated linear probability models of the following difference in differences specification^4,5^:

$y_{izt}=b_{0}+b_{1}{*Post}_{t}+b_{2}{*MA}_{iz}+b_{3}\left( {Post}_{t}*{MA}_{ih} \right)+b_{4}*x_{i}+u_{t}+v_{z}+e_{izt}$ (1)

where $y_{izt}$ is a dichotomous indicator of transportation to the reference ED for patient $i$ in zip code $z$ and time period $t$ (year). ${Post}_{t}$ is a dichotomous indicator of the post-ban period (2009 and later); ${MA}_{iz}$ is an indicator that takes value 1 if patient $i$ is from MA (exposed) and value 0 otherwise (unexposed); $x_{i}$ indicates individual characteristics (age, sex, race, comorbidities, dual Medicaid coverage); $u_{t}$ are dichotomous indicators of unobserved secular changes in the outcome measure (year fixed effect), and $z$ are dichotomous indicators of unobserved differences by zip code in factors that affect the outcome measure (zip code fixed-effect).^4^ The coefficient of the interaction term ${Post}_{t}*{MA}_{iz}$ gives the percentage change in the outcome measure associated with AD ban. A negative estimate indicates a percentage reduction in the outcome rate (e.g., proportion transported to reference ED), while a positive estimate indicates an increase. We obtained standard errors clustered at the state level to mitigate underestimation due to unobserved systematic differences by area level.^6,7^ We assessed statistical significance at the 5 percent level.

**eFigure 1. Most frequent ED/hospital among EMS transported White patients in each zip code in Boston**


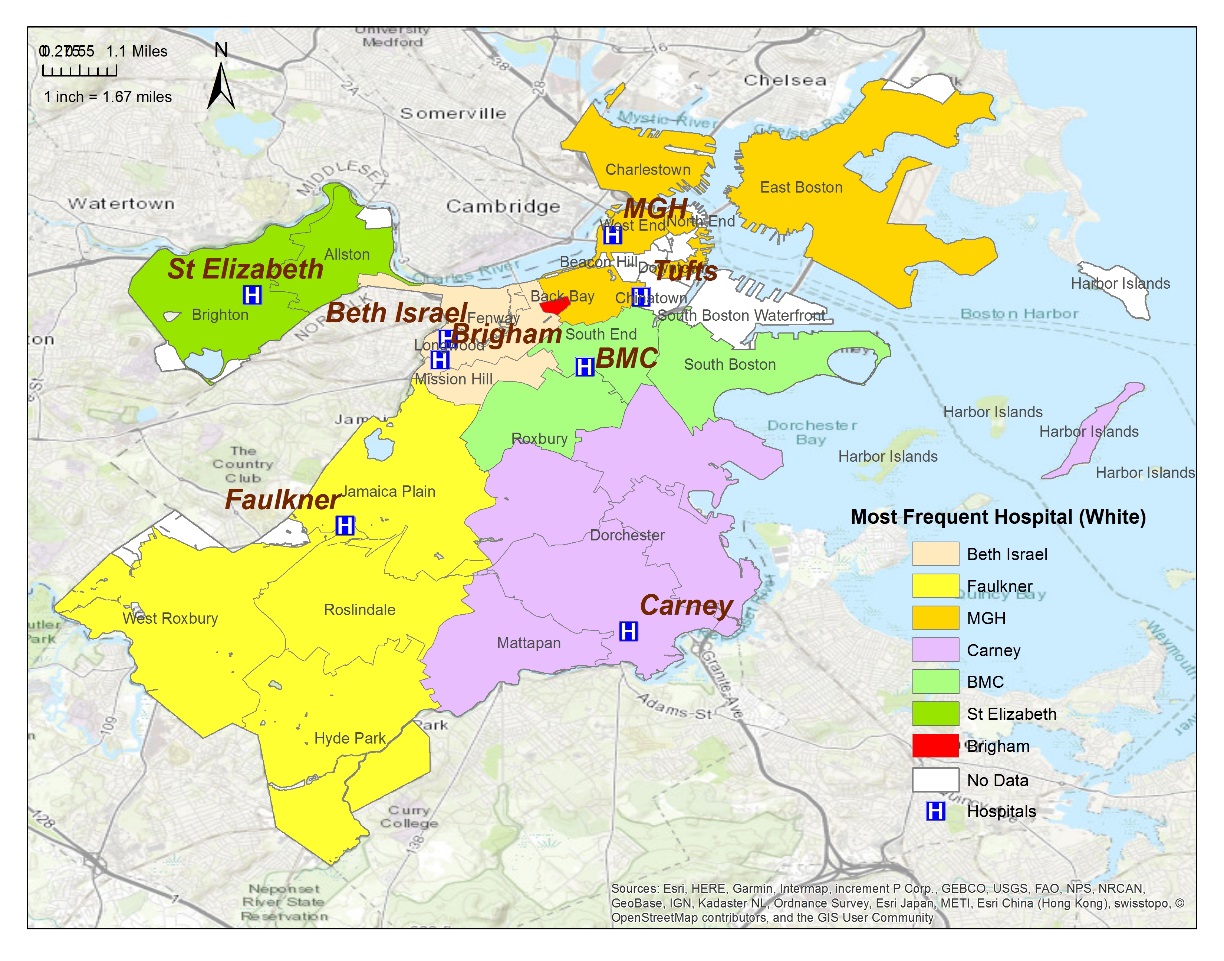


Notes:

1) The boundaries reflect zip code areas. Some zip codes (marked "No Data", in white color) did not have sufficient number of Medicare enrollees of each race/ethnicity.

2) The color coding is by reference ED/hospital. So, all yellow zip codes are where the most common destination among non-Hispanic White patients (residing in these zip codes) was to Faulkner Hospital.

eTable 6. Prevalence of chronic conditions at baseline

| Chronic Condition Comorbidity, % | All | Massachusetts | | Other states | |
| --- | --- | --- | --- | --- | --- |
|  |  | Pre-ban | Post-ban | Pre-ban | Post-ban |
| Alzheimer's disease, dementia | 40.7% | 33.9% | 37.0% | 38.9% | 41.9% |
| Atrial fibrillation | 24.1% | 25.5% | 25.6% | 22.8% | 24.5% |
| Chronic kidney disease | 39.0% | 36.3% | 42.4% | 32.8% | 41.4% |
| COPD | 32.9% | 31.2% | 30.8% | 33.1% | 33.0% |
| Heart failure | 49.2% | 46.6% | 45.4% | 49.4% | 49.5% |
| Diabetes | 45.1% | 40.6% | 41.7% | 44.8% | 45.6% |
| Hip / pelvic fracture | 6.4% | 5.3% | 5.1% | 7.0% | 6.2% |
| Ischemic heart disease | 66.2% | 65.6% | 58.3% | 66.8% | 66.4% |
| Depression | 35.6% | 36.3% | 39.0% | 32.4% | 36.9% |
| Rheumatoid arthritis / osteoarthritis | 53.2% | 44.5% | 48.3% | 49.9% | 54.9% |
| Stroke / transient ischemic attack | 19.0% | 14.2% | 13.0% | 21.0% | 18.5% |
| Cancer, breast | 4.8% | 5.6% | 5.6% | 4.4% | 4.9% |
| Cancer, colorectal | 2.9% | 3.0% | 4.1% | 2.3% | 3.0% |
| Cancer, prostate | 4.6% | 4.9% | 4.4% | 4.3% | 4.7% |
| Cancer, lung | 1.7% | 2.1% | 2.3% | 1.5% | 1.8% |
| Cancer, endometrial | 0.6% | 0.6% | 0.7% | 0.6% | 0.6% |
| Anemia | 60.9% | 53.8% | 57.3% | 59.3% | 61.7% |
| Asthma | 12.1% | 12.2% | 12.8% | 11.1% | 12.5% |
| Hyperlipidemia | 63.9% | 55.6% | 65.3% | 57.4% | 66.3% |
| Benign prostatic hyperplasia | 11.3% | 9.8% | 10.5% | 9.3% | 12.1% |
| Hypertension | 90.3% | 87.9% | 89.0% | 88.6% | 91.1% |
| Acquired hypothyroidism | 26.5% | 18.6% | 23.7% | 24.1% | 27.7% |

eTable 7. Comparison of average distance between first and second most frequent destination

|  | Distance to ED, miles: Mean (Std. Dev.) | | |
| --- | --- | --- | --- |
|  | First most frequent destination ED ("reference ED") | Second most frequent destination ED | Difference  [95% confidence interval] |
| All zip codes | 4.8 (4.1) | 6.4 (5.5) | 1.62 [1.58, 1.66] |
| Subgroups of zip codes by # hospitals EDs in 10-mile vicnity | | | |
| 0 to 1 | 5.1 (4.2) | 7.1 (5.7) | 1.98 [1.93, 2.03] |
| 2 or more | 2.8 (2.3) | 3.6 (2.9) | 0.78 [0.74, 0.83] |
| Urban location | | | |
| Zip code in largest 16 cities | 3.4 (3.0) | 4.3 (3.6) | 0.88 [0.82, 0.95] |

Notes:

1) Using data on mileage of the EMS transport reported on the claims record, here we compared the mileage for the transports to the reference ED (most frequent) with the mileage to the transports to the second most frequent ED destination, all among patients from the same zip code. Distance reported for patients from same zip code can vary due to difference in street pick-up location.

2) For the estimates reported in the table we included only the EMS transports to either the first or second most frequent destination ED. The mean and standard deviation are observed values for each subgroup of transports. The difference estimates reported are based on a linear regression of mileage distance with zip code fixed effects and indicator of second most frequent destination as the covariate.

eTable 8. Estimates of the impact of ban on transport to reference ED: All and by race/ethnicity

| Covariate | Model: All | | Model: Interaction by race/ethnicity | |
| --- | --- | --- | --- | --- |
|  | Coefficient | 95% Confidence Interval | Coefficient | 95% Confidence Interval |
| Reference cohort rate | 0.5506 | [0.5114,0.5898] | 0.5515 | [0.5129,0.5901] |
| Transition period | 0.0062 | [-0.0151,0.0276] | 0.007 | [-0.0167,0.0306] |
| Massachusetts x Transition Period | 0.0058 | [-0.0093,0.0210] | 0.0062 | [-0.0113,0.0237] |
| Post period | 0.0095 | [-0.0057,0.0246] | 0.0078 | [-0.0116,0.0272] |
| Massachusetts x Post Period | -0.027 | [-0.0404,-0.0135] | -0.0275 | [-0.0451,-0.0099] |
| Massachusetts x Black |  |  | 0.0394 | [0.0101,0.0687] |
| Massachusetts x Hispanic |  |  | 0.0441 | [0.0253,0.0630] |
| Transition x Black |  |  | -0.0213 | [-0.0438,0.0013] |
| Transition x Hispanic |  |  | 0.0167 | [-0.0085,0.0418] |
| Massachusetts x Transition x Black |  |  | 0.0248 | [0.0027,0.0469] |
| Massachusetts x Transition x Hispanic |  |  | -0.0182 | [-0.0438,0.0074] |
| Post period x Black |  |  | 0.0162 | [-0.0153,0.0476] |
| Post period x Hispanic |  |  | -0.0058 | [-0.0330,0.0214] |
| Massachusetts x Post x Black |  |  | -0.0131 | [-0.0449,0.0187] |
| Massachusetts x Post x Hispanic |  |  | 0.0365 | [0.0094,0.0636] |
| Rac/ethnicity (reference White) |  |  |  |  |
| Black | -0.0898 | [-0.1135,-0.0662] | -0.0997 | [-0.1302,-0.0693] |
| Hispanic | -0.061 | [-0.0836,-0.0385] | -0.0596 | [-0.0807,-0.0385] |
| Age (reference 66-74) |  |  |  |  |
| 75-84 | 0.028 | [0.0122,0.0438] | 0.028 | [0.0122,0.0437] |
| 85+ | 0.0396 | [0.0216,0.0576] | 0.0396 | [0.0217,0.0575] |
| Female | 0.0168 | [0.0051,0.0286] | 0.0169 | [0.0051,0.0286] |
| Dual Medicaid coverage | -0.0011 | [-0.0124,0.0102] | -0.0011 | [-0.0124,0.0102] |
| Principal ED diagnosis (reference AMI) |  |  |  |  |
| Congestive heart failure | 0.0051 | [-0.0493,0.0595] | 0.0052 | [-0.0493,0.0598] |
| Pneumonia | 0.0369 | [-0.0145,0.0883] | 0.037 | [-0.0143,0.0884] |
| Stroke | -0.0017 | [-0.0619,0.0584] | -0.0016 | [-0.0619,0.0586] |
| Sepsis | 0.0107 | [-0.0350,0.0564] | 0.0108 | [-0.0349,0.0565] |
| Gastrointestinal bleeding | 0.0029 | [-0.0590,0.0649] | 0.003 | [-0.0595,0.0655] |
| Arrhythmia | 0.0045 | [-0.0415,0.0506] | 0.0045 | [-0.0416,0.0507] |
| Serious injury/trauma | -0.0423 | [-0.0822,-0.0024] | -0.0423 | [-0.0823,-0.0024] |
| Other | 0.0123 | [-0.0312,0.0558] | 0.0123 | [-0.0313,0.0560] |
| Comorbidity |  |  |  |  |
| Alzheimer's disease, dementia | -0.0289 | [-0.0421,-0.0156] | -0.0288 | [-0.0421,-0.0156] |
| Atrial fibrillation | 0.0069 | [-0.0120,0.0258] | 0.0068 | [-0.0120,0.0257] |
| Chronic kidney disease | -0.0007 | [-0.0100,0.0087] | -0.0006 | [-0.0100,0.0088] |
| COPD | 0.0078 | [0.0001,0.0155] | 0.0078 | [-0.0000,0.0156] |
| Heart failure | 0.0161 | [0.0012,0.0309] | 0.0161 | [0.0013,0.0309] |
| Diabetes | 0.0079 | [-0.0041,0.0198] | 0.0078 | [-0.0041,0.0198] |
| Hip / pelvic fracture | 0.018 | [-0.0056,0.0417] | 0.0179 | [-0.0057,0.0415] |
| Ischemic heart disease | 0.0016 | [-0.0171,0.0203] | 0.0017 | [-0.0170,0.0203] |
| Depression | -0.0016 | [-0.0133,0.0101] | -0.0016 | [-0.0133,0.0102] |
| Rheumatoid arthritis / osteoarthritis | 0.0042 | [-0.0095,0.0180] | 0.0043 | [-0.0095,0.0180] |
| Stroke / transient ischemic attack | 0.0072 | [-0.0069,0.0212] | 0.0072 | [-0.0068,0.0212] |
| Cancer, breast | -0.0026 | [-0.0268,0.0215] | -0.0027 | [-0.0268,0.0214] |
| Cancer, colorectal | -0.0049 | [-0.0454,0.0357] | -0.0048 | [-0.0453,0.0357] |
| Cancer, prostate | 0.0075 | [-0.0147,0.0297] | 0.0074 | [-0.0149,0.0297] |
| Cancer, lung | -0.0061 | [-0.0461,0.0338] | -0.006 | [-0.0463,0.0342] |
| Cancer, endometrial | -0.0175 | [-0.1133,0.0784] | -0.0174 | [-0.1133,0.0785] |
| Anemia | -0.0042 | [-0.0162,0.0079] | -0.0041 | [-0.0162,0.0080] |
| Asthma | 0.0204 | [0.0009,0.0399] | 0.0204 | [0.0009,0.0398] |
| Hyperlipidemia | 0.0082 | [-0.0045,0.0210] | 0.0082 | [-0.0046,0.0210] |
| Benign prostatic hyperplasia | 0.0025 | [-0.0175,0.0224] | 0.0025 | [-0.0174,0.0225] |
| Hypertension | 0.0287 | [0.0167,0.0408] | 0.0288 | [0.0167,0.0408] |
| Acquired hypothyroidism | 0.0047 | [-0.0139,0.0233] | 0.0047 | [-0.0139,0.0234] |

Notes:

1) Not reported are calendar month and year indicators

eTable 9. Parallel trends test results

| Patient cohort | Change associated with MA AD ban, percentage points  [95% confidence interval] | |
| --- | --- | --- |
|  | % EMS transports to reference ED | % transported to safety-net ED |
| All | -0.4 [-1.6, 0.7] | -0.2 [-1.7, 1.2] |
| Race/ethnicity |  |  |
| White patients, non-Hispanic | 0.4 [-1.0, 1.7] | -0.1 [-1.8, 1.5] |
| Black patients, non-Hispanic | -1.1 [-4.1, 2.0] | 0.8 [-0.8, 2.5] |
| Hispanic patients | **-2.5 [-4.8, -0.2]** | -1.1 [-3.8, 1.7] |

eTable 10. Sensitivity 1 - Select only the top 10 states

| Patient cohort | % EMS transports to reference ED in Massachusetts | | | % EMS transports to reference ED in comparison states | | | Unadjusted relative change (percentage points) | Adjusted relative change, percentage points [95% CI] | p-value |
| --- | --- | --- | --- | --- | --- | --- | --- | --- | --- |
|  | Pre-ban | Post-ban | percentage point change | Pre-ban | Post-ban | percentage point change |  |  |  |
| All | 65.1% | 63.8% | -1.3 | 59.6% | 62.0% | 2.4 | -3.7 | **-3.2 [-4.7, -1.8]** | 0.001 |
| Race/ethnicity |  |  |  |  |  |  |  |  |  |
| White patients, non-Hispanic | 67.2% | 66.0% | -1.2 | 62.9% | 65.4% | 2.5 | -3.7 | **-3.5 [-5.4, -1.5]** | 0.003 |
| Black patients, non-Hispanic | 43.6% | 41.8% | -1.8 | 45.6% | 48.7% | 3.1 | -4.9 | **-4.0 [-6.6, -1.4]** | 0.007 |
| Hispanic patients | 62.5% | 62.2% | -0.3 | 52.1% | 51.8% | -0.4 | 0.1 | 0.9 [-1.5, 3.2] | 0.423 |

Notes

1) Observed % EMS transport rates are adjusted for sampling weights

2) From the data for the main analysis we only retained the top 10 comparison states by number of EMS transports: CA TX FL NY NJ IL MI PA NC GA

eTable 11. Sensitivity 12- Select only the top 5 states

| Patient cohort | % EMS transports to reference ED in Massachusetts | | | % EMS transports to reference ED in comparison states | | | Unadjusted relative change (percentage point) | Adjusted relative change, percentage point [95% CI] | p-value |
| --- | --- | --- | --- | --- | --- | --- | --- | --- | --- |
|  | Pre-ban | Post-ban | percentage point change | Pre-ban | Post-ban | percentage point change |  |  |  |
| All | 65.1% | 63.8% | -1.3 | 58.7% | 60.5% | 1.8 | -3.1 | **-2.6 [-4.5, -0.7]** | 0.016 |
| Race/ethnicity |  |  |  |  |  |  |  |  |  |
| White patients, non-Hispanic | 67.2% | 66.0% | -1.2 | 62.2% | 63.9% | 1.7 | -2.9 | **-2.6 [-5.2, -0.007]** | 0.054 |
| Black patients, non-Hispanic | 43.6% | 41.8% | -1.8 | 45.1% | 48.3% | 3.2 | -5.0 | **-4.2 [-7.2, -1.3]** | 0.015 |
| Hispanic patients | 62.5% | 62.2% | -0.3 | 52.1% | 51.6% | -0.5 | 0.2 | 1.2 [-1.8, 4.3] | 0.352 |

Notes

1) Observed % EMS transport rates are adjusted for sampling weights

2) From the data for the main analysis we only retained the top 10 comparison states by number of EMS transports: CA TX FL NY NJ

eTable 12. Sensitivity 13- Exclude 2007 cases

| Patient cohort | % EMS transports to reference ED in Massachusetts | | | % EMS transports to reference ED in comparison states | | | Unadjusted relative change (percentage point) | Adjusted relative change, percentage point [95% CI] | p-value |
| --- | --- | --- | --- | --- | --- | --- | --- | --- | --- |
|  | Pre-ban | Post-ban | percentage point change | Pre-ban | Post-ban | percentage point change |  |  |  |
| All | 64.9% | 63.8% | -1.1 | 61.8% | 62.7% | 0.9 | -2.0 | **-2.5 [-4.1, -0.9]** | 0.004 |
| Race/ethnicity |  |  |  |  |  |  |  |  |  |
| White patients, non-Hispanic | 67.0% | 66.0% | -1.0 | 64.8% | 65.9% | 1.1 | -2.1 | **-2.7 [-4.7, -0.7]** | 0.011 |
| Black patients, non-Hispanic | 44.9% | 41.8% | -3.1 | 47.2% | 49.3% | 2.1 | -5.1 | **-6.8 [-9.7, -3.9]** | <0.001 |
| Hispanic patients | 60.4% | 62.2% | 1.9 | 56.5% | 52.2% | -4.3 | 6.2 | **5.5 [4.1, 6.9]** | <0.001 |

Notes

1) Observed % EMS transport rates are adjusted for sampling weights

2) From the data for the main analysis we excluded the 2007 cases. So the pre-ban cases are from 1/1/2008 to 6/30/2008.

**References**

1. Agency for Healthcare Research and Quality. Medical Expenditure Panel Survey2020, Bethesda, MA.

2. Centers for Medicare & Medicaid Services. *Medicare Current Beneficiary Survey (MCBS).* <https://www.cms.gov/Research-Statistics-Data-and-Systems/Research/MCBS/>: Centers for Medicare & Medicaid Services;2016.

3. Korn EL, Graubard BI. *Analysis of health surveys.* Vol 323: John Wiley & Sons; 2011.

4. Ryan AM, Burgess JF, Jr., Dimick JB. Why We Should Not Be Indifferent to Specification Choices for Difference-in-Differences. *Health Serv Res.* 2015;50(4):1211-1235.

5. Wing C, Simon K, Bello-Gomez RA. Designing Difference in Difference Studies: Best Practices for Public Health Policy Research. *Annual Review of Public Health.* 2018;39(1):453-469.

6. Cameron AC, Trivedi PK. *Microeconometrics: Methods and Applications.* New York: Cambridge University Press; 2005.

7. Bertrand M, Duflo E, Mullainathan S. How much should we trust differences-in-differences estimates? *Quarterly Journal of Economics.* 2004;119(1):249-275.
